# A Holistic Look at Minimizing Adverse Environmental Impact Under Section 316(b) of the Clean Water Act

**DOI:** 10.1100/tsw.2002.183

**Published:** 2002-04-18

**Authors:** John A. Veil, Markus G. Puder, Debra J. Littleton, Nancy Johnson

**Affiliations:** Argonne National Laboratory, 955 L’Enfant Plaza, SW, Suite 6000, Washington, DC 20024, USA; U.S. Department of Energy, Office of Fossil Energy, 1000 Independence Avenue, SW, Washington, DC 20585, USA

**Keywords:** cooling water, intake structure, adverse environmental impact, 316(b), entrainment, impingement

## Abstract

Section 316(b) of the Clean Water Act (CWA) requires that “the location, design, construction, and capacity of cooling water intake structures reflect the best technology available for minimizing adverse environmental impact.” As the U.S. Environmental Protection Agency (EPA) develops new regulations to implement Section 316(b), much of the debate has centered on adverse impingement and entrainment impacts of cooling-water intake structures. Depending on the specific location and intake layout, once-through cooling systems withdrawing many millions of gallons of water per day can, to a varying degree, harm fish and other aquatic organisms in the water bodies from which the cooling water is withdrawn. Therefore, opponents of once-through cooling systems have encouraged the EPA to require wet or dry cooling tower systems as the best technology available (BTA), without considering site-specific conditions.

However, within the context of the broader scope of the CWA mandate, this focus seems too narrow. Therefore, this article examines the phrase “minimizing adverse environmental impact” in a holistic light. Emphasis is placed on the analysis of the terms “environmental” and “minimizing.” Congress chose “environmental” in lieu of other more narrowly focused terms like “impingement and entrainment,” “water quality,” or “aquatic life.” In this light, BTA for cooling-water intake structures must minimize the entire suite of environmental impacts, as opposed to just those associated with impingement and entrainment. Wet and dry cooling tower systems work well to minimize entrainment and impingement, but they introduce other equally important impacts because they impose an energy penalty on the power output of the generating unit. The energy penalty results from a reduction in plant operating efficiency and an increase in internal power consumption. As a consequence of the energy penalty, power companies must generate additional electricity to achieve the same net output. This added production leads to additional environmental impacts associated with extraction and processing of the fuel, air emissions from burning the fuel, and additional evaporation of freshwater supplies during the cooling process. Wet towers also require the use of toxic biocides that are subsequently discharged or disposed. The other term under consideration, “minimizing,” does not equal “eliminating.” Technologies may be available to minimize but not totally eliminate adverse environmental impacts.

### INTRODUCTION

The U.S. Environmental Protection Agency's (EPA's) rationale for proposing rigorous new-facility intake structure requirements was based on the agency's desire to minimize the number of aquatic organisms that is trapped on an intake structure during cooling-water withdrawal (impinged) or carried by the cooling-water flow through the entire cooling system (entrained). While impingement and entrainment are real environmental impacts, some stakeholders in the regulatory process have viewed these impacts as the only basis for decision making[1,2]. Some of the alternative technologies to once-through cooling (e.g., wet and dry cooling towers) are extremely effective at minimizing impingement and entrainment impacts, but their use introduces other types of adverse environmental impacts (AEIs). This article develops a broader, more holistic concept of AEIs: impingement, entrainment, as well as several others.

Some stakeholders have postulated that cooling towers are not part of cooling-water intake structures and should therefore not even be considered as regulatory options under Section 316(b). The following discussion deals with minimizing AEIs rather than a full interpretation of Section 316(b). Therefore, the discussion does not enter into the debate about whether requiring cooling towers is an appropriate regulatory option.

Much of the discussion contained in the following sections was gleaned from the years of active debate surrounding the Section 316(b) issue. The authors have previously raised some of the points presented here, while others have been taken from the extensive public record that has been presented to the EPA during several public meetings and open comment periods.

### POWER PLANT COOLING SYSTEMS

In 1999, more than 60% of the utility power-generating capacity in the U.S. (382,270 MW) utilized the steam electric process[3]. At nuclear and fossil-fuel power plants, electricity is produced by heating purified water to create high-pressure steam. The steam is expanded in turbines, which drive the generators that produce electricity. After leaving the turbines, the steam passes through a condenser with multiple tubes and a large surface area. A high volume of cool water circulates through the tubes, absorbing heat from the steam. As the steam cools and condenses, the temperature of the cooling water rises.

Most power plants use either once-through cooling or closed-cycle cooling. Once-through cooling systems withdraw large volumes of water, typically in the range of tens of millions to billions of gallons per day, from a river, lake, estuary, or ocean. The water is pumped through the condenser and finally returned to the same or a nearby water body. Closed-cycle cooling systems recirculate cooling water to a cooling tower and basin, cooling pond, or cooling lake before returning it to the condenser. Because evaporation and planned cooling-tower blowdown remove cooling water from the evaporative system, regular additions of “makeup” cooling water are needed. At many plants, the makeup water is withdrawn from surface water bodies. Makeup volumes are much lower than daily once-through volumes and may range from hundreds of thousands to millions of gallons per day.

The most commonly used type of closed-cycle cooling systems employs wet cooling towers, where water rejects heat to the atmosphere through evaporation and sensible heat transfer to the ambient air flowing through the tower. The air flow through the tower is maintained by fans (mechanical draft) or by convective currents created by the shape of the tower (natural draft).

Some stakeholders have advocated the dry cooling tower method because it requires even less makeup water than a wet tower. Dry towers remove heat to the atmosphere only by sensible heat transfer. They do not rely on evaporation and, therefore, require little makeup water. Few dry towers have been installed in power-plant-sized applications (typically such units have a capacity of several hundred megawatts) to date because of cost and practical thermodynamic heat transfer limitations.

### LEGAL AND REGULATORY CONSIDERATIONS

#### Section 316(b) of the Clean Water Act

Steam electric power plants and other industries that withdraw cooling water from surface water bodies (e.g., pulp and paper, iron and steel, chemical, manufacturing, petroleum refineries, and offshore oil and gas production) must comply with the terms of Section 316(b) of the Clean Water Act (CWA) enacted by Congress in 1972:

> "Any standard established pursuant to Section 301 or Section 306 of this Act and applicable to a point source shall require that the location, design, construction, and capacity of cooling water intake structures reflect the best technology available for minimizing adverse environmental impact."

This single statutory sentence has spawned numerous biological studies and technological and operational plant modifications. The cost of these actions has been very high.

### Implementation of Section 316(b)

In 1976, the EPA promulgated final Section 316(b) regulations (April 26, 1976; 41 FR 17387). However, these regulations were successfully challenged by a group of 58 utilities [*Appalachian Power Co. v. Train*, 10 ERC 1965 (4th Cir. 1977)]. In 1979, the EPA formally withdrew its Section 316(b) regulations (June 1979; 44 FR 32956). As a consequence of the vacuum created by the absence of more-detailed federal regulations, states implemented Section 316(b) in different ways. The broad statutory language facilitated widely differing interpretations by the states. Some adopted comprehensive programs, others imposed less rigorous requirements, and some never developed any formal regulations.

In the mid-1990s, a coalition of environmental groups, headed by the Hudson Riverkeeper, filed suit against the EPA over failure to repromulgate Section 316(b) regulations [*Cronin et al. v. Reilly*, 93 Civ. 0314 (AGS)]. On October 10, 1995, the U.S. District Court, Southern District of New York, entered a Consent Decree between the parties directing the EPA to regulate cooling-water intake structures. Under the Consent Decree, the EPA agreed to propose regulations by June 1999 and promulgate a final rule by 2001. The Consent Decree was modified on November 21, 2000, to: (a) take final action on new facility regulations by November 9, 2001; (b) propose existing source utility and nonutility power producer regulations by February 28, 2002, and take final action on those regulations by August 28, 2003; and (c) propose regulations for other existing facilities not covered in (b) above by June 15, 2003, and take final action on those regulations by December 15, 2004.

### Legislative History

The language of Section 316(b) is quite short and easily subject to wide interpretation. Legislative history may prove to be a powerful tool for ascertaining the congressional intent behind Section 316(b). Legislative history consists of all legislative events that transpired throughout the process of enacting or defeating proposed legislation. The documentation generally includes the bill, the drafts, and the preceding markup documents; the testimony at hearings; the reports or studies commissioned by the legislature; the chronology of voting; the floor debates; and the message of the executive accompanying the signature or veto of the bill.

Little legislative history exists with respect to Section 316(b). A recent law journal article discusses this history[4]. The language appeared suddenly, without any further explanation, in the Federal Water Pollution Control Act (the law that was modified in 1972 to become the CWA) Conference Report under the heading “Regulation of Thermal Discharges” in “Title III—Standards and Enforcement.” The Conference Report provided no additional commentary but merely included the language that was to become law. Neither the Senate nor House bill had included intake-structure language like the Section 316(b) language that the conference committee added.

Except for one contribution, subsequent floor debates failed to shed more light on the dynamics that led to the conference substitute. Representative Clausen, during the House consideration of the Conference Report, summarized Section 316(b) in his own words. In departure from the actual conference language, he used the word “any” in connection with “adverse environmental impact.” He added that “the reference [in Section 316(b)] ... to ‘best technology available’ is intended to be interpreted to mean the best technology available at an economically practicable cost[5].”

Section 316(b) has never been amended. Although reform bills were periodically introduced over the years, no legislation has been passed. Thus, the minimalist language of Section 316(b) remains as initially enacted.

### Interpretation of Section 316(b) Language

The following sections discuss and analyze two statutory terms that are central to Section 316(b): “adverse environmental impact” and “minimizing.”

### Adverse Environmental Impact

“Adverse” denotes undesirable attributes. In the context of Section 316(b), adverse has often been interpreted as relating to the extent of biological harm through impingement or entrainment. However, some agencies and other stakeholders have also considered increased quantities of an air or water pollutant or the amount of water lost through evaporation to represent adverse circumstances. Adverse is not an absolute but a subjective term that should be assessed in relation to the entire system or universe under review. For example, does the addition of 5 kg/day of nitrogen to a water body cause an effect that could be considered an adverse impact? If the water body were a small pond or slow-flowing stream, the nitrogen addition would likely cause an undesirable adverse effect. If the nitrogen entered a large river or an estuary with a total nutrient budget of thousands of kilograms of nitrogen, the consequences (if any) would be small. No adverse impacts would be expected in this latter case. Another example involves entrainment loss of fish larvae. In a small water body that hosts limited populations of important species, the entrainment of 1,000 larvae might have a critical adverse impact. However, in a large water body with extensive populations of species, the loss of 1,000 larvae would be insignificant to the species population, the food chain, and the overall ecosystem. In this light, the entrainment would not pose an adverse impact.

The other relevant word in Section 316(b) is “environmental.” In a regulatory context, the term encompasses a wide range of attributes of the natural world, including air, water, land, noise, and their relationship to one another. The statutory definitions provided in major environmental laws support this broad reading. Section 101 of the Comprehensive Environmental Response, Compensation, and Liability Act defines the term “environment” as “surface water, ground water, drinking water supply, land surface or subsurface strata, or ambient air.” Under Section 329 of the Emergency Planning and Community Right-to-Know Act and Section 3(5) of the Toxic Substances Control Act, “The term ‘environment’ includes water, air, and land and the interrelationship which exists among and between water, air, and land and all living things.”

In the Section 316(b) debates of recent years, however, the EPA has confined “environment” to aquatic organisms and focused just on impingement and entrainment. These are very real impacts that are attributable to water intakes. However, the CWA mandates are not well-served by this narrow focus. In fact, a whole suite of environmental impacts is associated with any type of cooling-water intake or cooling-water system. For each intake or system, the relative importance of each type of impact will vary. A comprehensive impact determination should consider the composite and cumulative impacts, along with any benefit offered by a particular cooling-water intake system. This article discusses important environmental impacts and describes their relative weight for once-through cooling systems, wet cooling tower systems, and dry cooling tower systems.

### Minimizing

“Minimize” and the related terms “minimum” and “minimal” appear throughout the CWA and its implementing regulations but are never defined. In the absence of a formal statutory or regulatory definition, two approaches can be used for ascertaining the meaning of the term. Consulting a dictionary provides a sense of the common usage of the word. Courts often use this approach to ascertain the plain meaning of terms. “Minimize” is defined in one dictionary as “to reduce to the smallest possible amount, extent, size, or degree[6].” Another dictionary defines the term as “to reduce or keep to a minimum” and defines “minimum” as “the least quantity assignable, admissible, or possible[7].” Neither definition implies complete elimination.

A second approach involves searching for the context and usage of the term in existing laws and regulations. The phrase “at a minimum” is commonly used in the CWA regulations and implies that the expected value for the parameter in question is a defined value greater than zero. Another example can be found on page 28869 of the EPA’s recent Notice of Data Availability for the Section 316(b) regulations (May 25, 2001; 66 FR 28853). The relevant text states that “EPA would not define these technologies [closed-cycle cooling and extremely low approach velocities] as BTA for ‘minimizing’ adverse environmental impact but instead determine that they avoid adverse environmental impact altogether.” In this example, the EPA emphasizes the difference between a technology that minimizes impacts and one that avoids or eliminates impacts entirely.

The concept of minimizing entails making a value judgment relative to the smallest amount of effect that is possible or acceptable. At each installation that falls under the purview of Section 316(b), it is necessary for accuracy and optimal decision making to make a site-specific evaluation of the relevant factors that may contribute to AEI.

During the past few years, the EPA has evaluated various types of technologies that can effectively reduce impingement and entrainment impacts (e.g., wedge wire screens, fine-mesh traveling screens with a fish return system, Gunderbooms). The degree of impact reduction achieved by these technologies varies at different locations, but in some instances they may perform well enough to be construed as effectively minimizing AEIs.

During the EPA’s Section 316(b) public meetings, some stakeholders have offered that the threshold for minimization should be “one dead fish equals adverse environmental impact.” The evidence presented in the previous paragraphs demonstrates that the loss of one fish does not equate to AEI.

## TYPES OF ADVERSE ENVIRONMENTAL IMPACTS

### Impingement and Entrainment

As a general rule, as more water is withdrawn from a surface water body, more aquatic organisms are likely to be impinged on the intake structure and entrained through the cooling-water system. Following this assumption, in most cases a cooling system that reduces the volume of water withdrawn will concomitantly reduce the number of organisms injured or killed by impingement and entrainment. Because this relationship is not linear (e.g., 10 times less flow does not necessarily result in 10 times less impingement and entrainment), the actual effects must be evaluated on a site-specific basis. Moreover, not all impinged and entrained organisms are killed or otherwise removed from the ecosystem. Nevertheless, the adverse impact associated with impingement and entrainment will usually be reduced when less cooling water is withdrawn. The highest impact in this category is associated with once-through cooling systems. Power plant once-through cooling systems typically withdraw in the range of tens of millions to billions of gallons per day. The volume of makeup water required for wet cooling towers is many times less than that used in once-through systems. The percentage reduction varies but generally falls in the range of 1% to more than 10% of the once-through flow volume. Dry towers use even less cooling water than wet cooling towers.

Cooling systems that rely on moving large volumes of air by fans—mechanical-draft wet towers and dry towers—may create their own form of impingement and entrainment. Insects and birds can be drawn into the intake plumes of large fans. Larger organisms can be trapped on the exterior of the fans or their intake coverings like insects caught on automobile radiators. Smaller organisms can be pulled through the moving fan and injured or killed. The authors are not aware of any published literature quantifying this impact, but the parallels to aquatic impingement and entrainment are obvious. Potentially, large batteries of fans may inflict harm to local populations of endangered insects or birds or important pollinator species. To place this in perspective, a dry tower installed to cool a power plant-sized unit might have banks of fans that cover several acres. For example, a dry tower system at a 40-MW geothermal power plant near Reno, Nevada, employs 240 fans covering a large surface area. Photographs of that facility are available at http://home.nvbell.net/sbgeo/steamboat.html .

Impingement and entrainment, when they result in death or harm to an organism, create an adverse impact to that organism. However, they do not necessarily create an adverse impact on the population or ecosystem at large. The principle of compensation—enhanced reproductive output by populations that have experienced loss of young members of the population—is well-documented in the literature. Compensation may serve to dampen the population-wide effects of impingement and entrainment. A thorough review of compensation in fish populations was submitted to the EPA by the Utility Water Act Group as part of its comments on the Section 316(b) new-facility proposed rule[8].

### Energy Penalty

Steam condensers are designed to produce a vacuum at the outlet end of the turbine, thereby increasing the efficiency of the system. The temperature of the cooling water exiting the condenser affects the performance of the turbine: the cooler the temperature, the better the performance. As cooling-water temperatures decrease, a higher level of vacuum can be produced and additional energy can be extracted. On an annual average, once-through cooling water has a lower temperature than recirculated water from a cooling tower. As a result of switching a once-through cooling system to a cooling tower, less energy can be generated by the power plant from the same amount of fuel. In addition, cooling towers use more electricity for pumps and fans than once-through systems. The net output of a plant that has converted its cooling system from a once-through system to a cooling tower will be reduced through both of these mechanisms. This reduction in energy output is known as the "energy penalty."

A 1992 report funded by the U.S. Department of Energy (DOE) found that the majority of literature values for the energy penalty associated with retrofitting fossil-fueled plants using once-through cooling with wet cooling towers were clustered in a band between 1.5% and 2.5%[9]. This means that a plant now equipped with a wet tower will produce 1.5 to 2.5% less electricity on an annual average than previously with a once-through system, while burning the same amount of fuel. Results for nuclear power plants showed greater variability, ranging between 1 and 5.8%. The data points were not as clearly clustered in a narrow range when compared with the data for the fossil-fuel plants. The authors of that report selected a range of 2 to 3% for the decrease in net electrical power that could be experienced if existing nuclear power plants retrofit from a once-through to a wet cooling system.

A report being prepared by the DOE, the National Energy Technology Laboratory (NETL), and the Argonne National Laboratory, scheduled to be completed in 2002, calculates the energy penalties that result from converting plants with once-through cooling to wet towers and to dry towers[10]. In a working draft of that analysis, the energy penalties are estimated for the hottest time of the summer months when electricity demand would also be at its peak by modeling hypothetical 400-MW coal-fired plants in five regions of the country with an ASPEN simulator model. The preliminary results indicate that conversion of a plant to a wet tower could cause energy penalties ranging from 2.4 to 4.0%. Conversion to an indirect dry tower, where possible, could cause energy penalties ranging from 8.9 to 12.1% under conservative design assumptions and 12.7% to almost 16% under a more realistic set of design assumptions. Annual average energy penalties will be smaller than those calculated for the hottest period of the year, but must still be considered.

The implications of the energy penalty are quite important from an environmental standpoint. To make up lost energy, additional fuel would have to be burned at the affected plants, at other plants within the power grid, or at completely new units that would have to be constructed. In addition, extraction and combustion of the additional fuel would cause several new AEIs.

#### *Fuel*

In general, the most probable type of generating unit used to make up the lost energy would be a fossil-fueled unit. The process of extracting fossil fuels creates impacts with respect to water and air, solid-waste generation, and land use. Moving the incremental fuel to the power plants consumes additional fuel for pumps or engines in transportation equipment.

#### *Air Emissions*

As fossil fuels are combusted, they give off a range of air pollutants of concern, including sulfur dioxide, nitrogen oxides, mercury, particulate matter, and carbon dioxide. It has been estimated that a plant converting from a once-through cooling system to a wet cooling tower increases its carbon dioxide emissions by 22 tons/year/MW for oil and gas and by 58 tons/year/MW for coal[11]. The DOE/NETL/Argonne report will also include estimates of annual energy penalties and air emissions. In addition to affecting air quality, some of the airborne pollutants may be deposited into surface water bodies, where they can directly affect aquatic organisms.

### Water Quality

Discharges of once-through cooling water and cooling tower blowdown to surface waters are subject to the conditions of National Pollutant Discharges Elimination System permits. Once-through cooling water may contain chlorine used as a biocide, but otherwise is unlikely to contain toxic chemicals added by the generating or the cooling processes. A wide range of toxic chemicals, including biocides (chlorine and other, more toxic chemicals), corrosion inhibitors, and scaling inhibitors, may be added to cooling towers[12]. Portions of the recirculating water in wet cooling tower systems are blown down periodically. These blowdown effluents contain residual levels of the toxic chemicals previously added to the towers. Dry towers will also have blowdown effluents, but the volume will be lower than for wet towers.

Heated discharges of once-through cooling water can have positive or negative effects. In temperate climates, warm discharges during the winter can enhance and sustain fish populations. At many locations, however, excessive heat can be damaging. Removal or addition of heat can yield different impacts on aquatic communities, depending on site-specific circumstances.

### Water Quantity and Consumption

The first type of water-quantity impact is related to availability of sufficient water for the cooling needs without causing adverse impacts on the water body and its other uses. Historically, power plants were sited near large bodies of water that could supply large volumes of once-through cooling water. As the use of wet cooling towers increased, new plants were also sited in locations where smaller volumes of water were available. Plants with dry towers need very little water and, from a water-supply perspective, can be sited in a wide range of locations. Plants cannot be sited on water bodies where cooling-water withdrawal could reduce the water level in the water body to a point where significant habitat was lost or water-quality conditions became undesirable. Plants using once-through cooling systems withdraw large volumes of water, although they return nearly all of that water to the water body at the same or a nearby location. Plants using cooling towers must also consider water availability, particularly if they are situated on a water body that is too small to support a withdrawal of several million gallons per day of makeup water that is not returned to the water body.

The second type of water-quantity impact relates to evaporation. Many freshwater bodies face heavy demands on their water. Any cooling system use that removes freshwater from those systems will need to compete with other existing and future uses. Plants with once-through cooling systems do not directly evaporate water. Virtually all water used for cooling is returned to the surface water body. However, the returned water is warm and will raise the temperature of the receiving water body to an extent that may increase the rate of natural evaporation from the surface of the water body. The Nuclear Regulatory Commission estimates that water lost by evaporation from a once-through-cooled discharge is about 60% of the evaporation from cooling towers[13].

In contrast, wet cooling tower systems intentionally evaporate water as an intrinsic part of the cooling process. Anecdotal accounts suggest that wet towers may have twice the evaporation rate of once-through cooling systems. One study estimates that when plants are converted from once-through cooling to cooling towers, an additional 15 gal/min of freshwater would be evaporated for each converted megawatt of generating capacity. This estimate assumes a conservative energy penalty of just 1%[11]. For a higher percentage energy penalty, the impacts would be proportionally higher.

Dry cooling towers do not rely on evaporation for cooling. Therefore, the evaporative losses associated with dry towers are very low.

### Solid Waste

Debris, trash, and other waterborne solids are captured by the bar racks at cooling-water intakes. The volume of solids corresponds to the volume of water that is passed through the intake structure. Therefore, once-through systems are likely to generate a larger volume of solid waste than closed-cycle systems. Wet towers accumulate sludge in the bottom of the towers that must be removed periodically. Dry towers collect some amount of airborne debris, including leaves and paper, on their intake screens. The relatively small solid-waste volumes attributable to all three types of systems are unlikely to cause AEIs.

### Noise

Noise emissions pose AEIs to workers, nearby residents, and wildlife. Like many other major industrial facilities, power plants can be noisy. Once-through cooling systems do not add appreciably to the overall background noise at power plants. However, cooling towers can create considerable noise levels. Natural-draft towers are noisy because of water movement through the tower. Mechanical-draft wet towers use banks of fans that contribute to plant noise levels, particularly in the vicinity of the towers themselves. Because of the large surface area needed to house the towers (up to several acres) and the large number of fans, dry towers are likely to be even noisier than the other types of cooling systems. Many jurisdictions have regulations controlling noise levels. The issue of noise could restrict the ability of some facilities to add banks of fans for cooling.

### Land Use/Habitat

It is difficult to assess what type of cooling system is likely to have the greatest adverse impact with respect to land use. Once-through systems need larger capacity intake structures that typically are built in the water body or on the shoreline. Some surface-water intakes require pipes that extend meters to kilometers offshore. Shore-side facilities for once-through systems are smaller than those for closed-cycle systems, however. The banks of towers, pumps, and piping used by closed-cycle systems may occupy significant land space.

Plants that convert from once-through systems to closed-cycle systems will consume more fuel. The process of extracting the fuel can disrupt terrestrial or aquatic habitats. As previously described, the energy penalty raises indirect land use/habitat concerns.

#### Plumes/Air Circulation

Once-through systems do not generate any significant plumes of water vapor and do not influence local air circulation. Wet towers release plumes that can contribute to deposition of undesirable particulates or salt onto surrounding land areas. Plants located in urban areas, near major highways, or in the flight paths of airports could create adverse impacts through fogging or icing conditions associated with plumes. These transportation-related impacts, though real, may not fall under the purview of "environmental impacts," depending on how broadly the term "environmental" is defined by a decision-making body. Such affects may be more correctly interpreted as safety impacts.

As a result of the large amount of air that must be moved through dry cooling systems, large banks of fans may affect local air circulation patterns. The authors are not aware of any research in this regard, but the issue could potentially affect windborne seed distribution and establish microclimates around the power plants. It is unknown whether these impacts would be adverse, positive, or neutral.

### DISCUSSION

The language of Section 316(b) is brief, leaving appreciable room for alternative interpretations. The authors believe that a holistic approach to Section 316(b) leads to the most rational interpretation. The preceding paragraphs suggest that the language of Section 316(b) directs regulatory decision makers to consider a wide range of AEIs. Moreover, the decision makers should evaluate how to minimize those impacts in the context of the physical and environmental setting of the power plant and the nearby water bodies. The evaluation should consider the cumulative impacts posed by all facilities, pollutant inputs, and natural processes operating in the water body.

The types of adverse impacts and the authors' qualitative assessment of their relative magnitude (high, moderate, low, or none) for each type of cooling system are summarized in Table 1. These rankings are not absolute and will vary somewhat depending on site-specific factors. However, Table 1 provides a useful consolidated presentation of the multiple types of impacts and reflects the discussion in the preceding sections. The authors believe that decision makers should undertake a comprehensive evaluation of all the factors listed in Table 1. Because this evaluation is qualitative, the different types of impacts summarized in Table 1 should not necessarily be given the same weight. For example, impingement and entrainment impacts are probably of greater significance than noise, plume, or air circulation impacts.

### EPA's Approach in the New-Facility Rules

The EPA proposed Section 316(b) rules for new sources on August 10, 2000 (65 FR 49060). Under the proposed rule, most new facilities would have had to install closed-cycle cooling systems (nearly all of these would use cooling towers). During public meetings and in written comments, the environmental community and several states have supported the use of dry cooling as the appropriate technology for addressing AEIs, while industry and several other federal agencies supported an approach involving site-specific evaluation.

**TABLE 1**
**Summary of Types of AEIs Associated with Cooling Systems**

| Type of AEI | Anticipated Magnitude of Impact* | | |
|---|---|---|---|
| | **Once-Through Cooling** | **Wet Cooling Tower** | **Dry Cooling Tower** |
| Impingement | H | L | L |
| Entrainment | H | L | L |
| Energy penalty | N | M to H | H |
| Additional air emissions | N | M to H | H |
| Additional fuel usage | N | M to H | H |
| Water quality | Heat: + or –<br>Biocides: L to M | Biocides: M to H | Biocides: L |
| Water consumption | L | M | M to H |
| Solid wastes | L | L | L |
| Noise | L | M | M to H |
| Land use/habitat | Aquatic: M to H<br>Terrestrial: L | Aquatic: L<br>Terrestrial: L to M | Aquatic: L<br>Terrestrial: M to H |
| Plumes | N | M to H | N |
| Air circulation | N | L | Unknown, but potentially M to H |

*Note*: Not all types of environmental impacts should be assigned the same weight.
*H = high, M = moderate, L = low, N = none.

The EPA published the final new-facility rules on December 18, 2001 (66 FR 65256). The new rules provide two alternative tracks. Under Track I, a fast track that does not consider actual site-specific environmental impacts, companies must meet a stringent set of rules, including installation of cooling towers or other equivalent flow-reduction measures, coupled with restrictions on the total flow volume, the ratio of intake flow volume to surface water body volume, and the intake velocity. In addition, plants are required to use other intake technologies. Since the process of obtaining permits becomes more predictable and timely, Track I may be attractive to many new facilities. Under Track II, companies must conduct intensive site-specific studies and projections at the facility in order to demonstrate to the permitting authority that an alternative set of intake controls will reduce impingement and entrainment to a level comparable to Track I controls. Approvals based on Track II would generally have a lower cost to applicants, but could make the permitting process more uncertain and lengthy.

The concept that AEI means more than just impingement and entrainment is captured in the EPA's requirements for Track II demonstrations at 40 CFR 125.84(d)(1)(i) and (ii):

> (i)This showing may include consideration of impacts other than impingement mortality and entrainment, including measures that will result in increases in fish and shellfish, but it must demonstrate comparable performance for species that the Director, in consultation with national, state or tribal fishery management agencies with responsibility for fisheries potentially affected by your cooling water intake structure, identifies as species of concern.
> (ii) In cases where air emissions and/or energy impacts that would result from meeting the requirements of paragraphs (b)(1) and (2) of this section would result in significant adverse impacts on local air quality, significant adverse impact on local water resources not addressed under paragraph (d)(1)(i) of this section, or significant adverse impact on local energy markets, you may request alternative requirements under Sec. 125.85.

### EPA's Upcoming Rules for Existing Facilities

The EPA is in the process of developing cooling-water intake regulations for existing utility and nonutility power-generating facilities but has not yet revealed any details of its proposed rule. There is a potential that at least some of the requirements in the proposal for new sources may be carried over to the rule for existing facilities. About 44% of the U.S. steam electric power plants employ once-through cooling systems[14]. If the final existing facility rule requires many or all of these plants to install dry or wet cooling tower systems, serious impacts with respect to electricity costs and availability could arise.

Moreover, such a decision could trigger other significant environmental impacts beyond impingement and entrainment, as described above.

### CONCLUSIONS

Congress added Section 316(b) to the CWA to ensure that cooling-water intakes did not cause unnecessary AEIs on water bodies. Impingement and entrainment can adversely impact aquatic ecosystems. However, decisions with respect to cooling-water intakes and systems made solely on the basis of minimizing or eliminating impingement and entrainment do not meet the CWA's comprehensive mandate and do not necessarily provide the best environmental protection. A holistic approach to Section 316(b) allows for a more thorough and comprehensive evaluation of the suite of potential AEIs associated with a cooling-water intake structure. It also allows for a comprehensive evaluation of whether a plant's environmental setting is being adversely impacted by a cooling-water intake structure.

### ACKNOWLEDGMENTS

Mr. Veil's and Dr. Puder's work was sponsored by the U.S. Department of Energy, Office of Fossil Energy, under contract W-31-109-Eng-38.
